# Novel candidate genes important for asthma and hypertension comorbidity revealed from associative gene networks

**DOI:** 10.1186/s12920-018-0331-4

**Published:** 2018-02-13

**Authors:** Olga V. Saik, Pavel S. Demenkov, Timofey V. Ivanisenko, Elena Yu Bragina, Maxim B. Freidin, Irina A. Goncharova, Victor E. Dosenko, Olga I. Zolotareva, Ralf Hofestaedt, Inna N. Lavrik, Evgeny I. Rogaev, Vladimir A. Ivanisenko

**Affiliations:** 1 Institute of Cytology and Genetics, Siberian Branch, Russian Academy of Sciences, Novosibirsk, Russia; 2 Research Institute of Medical Genetics, Tomsk NRMC, Tomsk, Russia; 3 Bogomoletz Institute of Physiology, Kiev, Ukraine; 4 Bielefeld University, International Research Training Group “Computational Methods for the Analysis of the Diversity and Dynamics of Genomes”, Bielefeld, Germany; 5 Bielefeld University, Technical Faculty, AG Bioinformatics and Medical Informatics, Bielefeld, Germany; 6 Department of Translational Inflammation, Institute of Experimental Internal Medicine, Otto von Guericke University, Magdeburg, Germany; 7 University of Massachusetts Medical School, Worcester, MA USA; 8 Department of Genomics and Human Genetics, Institute of General Genetics, Russian Academy of Sciences, Moscow, Russia; 9 Center for Genetics and Genetic Technologies, Faculty of Biology, Faculty of Bioengineering and Bioinformatics, Lomonosov Moscow State University, Moscow, Russia

**Keywords:** Comorbidity, Asthma, Hypertension, Apoptosis, Central nervous system, ANDSystem, Associative gene networks, Gene prioritization

## Abstract

**Background:**

Hypertension and bronchial asthma are a major issue for people’s health. As of 2014, approximately one billion adults, or ~ 22% of the world population, have had hypertension. As of 2011, 235–330 million people globally have been affected by asthma and approximately 250,000–345,000 people have died each year from the disease. The development of the effective treatment therapies against these diseases is complicated by their comorbidity features. This is often a major problem in diagnosis and their treatment. Hence, in this study the bioinformatical methodology for the analysis of the comorbidity of these two diseases have been developed. As such, the search for candidate genes related to the comorbid conditions of asthma and hypertension can help in elucidating the molecular mechanisms underlying the comorbid condition of these two diseases, and can also be useful for genotyping and identifying new drug targets.

**Results:**

Using ANDSystem, the reconstruction and analysis of gene networks associated with asthma and hypertension was carried out. The gene network of asthma included 755 genes/proteins and 62,603 interactions, while the gene network of hypertension - 713 genes/proteins and 45,479 interactions. Two hundred and five genes/proteins and 9638 interactions were shared between asthma and hypertension. An approach for ranking genes implicated in the comorbid condition of two diseases was proposed. The approach is based on nine criteria for ranking genes by their importance, including standard methods of gene prioritization (Endeavor, ToppGene) as well as original criteria that take into account the characteristics of an associative gene network and the presence of known polymorphisms in the analysed genes. According to the proposed approach, the genes IL10, TLR4, and CAT had the highest priority in the development of comorbidity of these two diseases. Additionally, it was revealed that the list of top genes is enriched with apoptotic genes and genes involved in biological processes related to the functioning of central nervous system.

**Conclusions:**

The application of methods of reconstruction and analysis of gene networks is a productive tool for studying the molecular mechanisms of comorbid conditions. The method put forth to rank genes by their importance to the comorbid condition of asthma and hypertension was employed that resulted in prediction of 10 genes, playing the key role in the development of the comorbid condition. The results can be utilised to plan experiments for identification of novel candidate genes along with searching for novel pharmacological targets.

**Electronic supplementary material:**

The online version of this article (10.1186/s12920-018-0331-4) contains supplementary material, which is available to authorized users.

## Background

Asthma is a chronic inflammatory disease of the respiratory tract, the main characteristics of which are hypersensitivity of the respiratory tract to various stimuli and reversible obstruction of airflow. The role of inflammation in the aetiology and pathogenesis of arterial hypertension is not so obvious upon first blush, but the significance of low-grade chronic inflammation in the development of metabolic syndrome, atherosclerosis, and obesity has been established in many studies [1–6]. It is known that the processes of coagulation and anti-coagulation, the fibrinolytic system, and thrombocytes are integral to asthma pathophysiology [7]. Therefore, in a number of studies, it was shown that asthma is associated with an increase in the incidence of cardiovascular diseases [8, 9]. As a consequence, the fact that the simultaneous diagnosis of asthma and arterial hypertension, which is termed comorbidity, in a high proportion of cases, is not surprising. According to Su et al. [10], the prevalence of hypertension in asthma patients (OR 1.66 [1.47, 1.88]; *P* < 0.00001) is lower only compared to cardiovascular, cerebrovascular, and obesity comorbidities. Apparently, this is not a coincidence as classic asthma mechanisms turned out to be a part of key processes of arterial hypertension initiation. A central example might be the discovery of the role of arachidonic acid-leukotriene B4 production in spontaneously hypertensive rats [11] or the significance of Th17 and IL17 in arterial hypertension [12]. Therapeutic treatment of allergic inflammation leads to improvement in the control of arterial pressure [13]. The importance of STAT3 transcription factors have also been uncovered - they participate in signal transduction with multiple cytokines and are active in allergic inflammation [14, 15] and vascular remodelling [16]. Based on these findings, it is possible to deduce that disturbance of the balance between pro-inflammatory and anti-inflammatory factors within the organism creates an optimal condition for implementation of the inherent propensity to both asthma and arterial hypertension. Besides the critical role of immune reactions and inflammation control in the pathogenesis of asthma and hypertension, other mechanisms are expected to be relevant in the comorbidity of these diseases. For example, β-adrenoblockers and ACE inhibitors are widely used to treat hypertension, but for a long time, they were contraindicated for patients with asthma because of the possibility of bronchoconstriction. In a large cohort of patients, it was demonstrated that adverse respiratory reactions to beta-blockers in the case of asthma partially depends on cardioselectivity, dose, and exposure duration [17]. Polymorphisms in b-adrenergic receptor genes are associated with the risk of hypertension and bronchial asthma [18–20]. It is assumed that mutations in the SLC26A4 gene can impact the pathogenesis of bronchial asthma and hypertension and, as such, the comorbidity of these diseases [21–24]. The SLC26A4 gene codes the pendrin protein with Cl-/HCO3- exchanger activity [25]. The loss of function of SLC26A4 in mice prevents development of bronchial asthma and hypertension symptoms; there is a possibility that mutations in the SLC26A4 gene among humans is a factor in the absence of these diseases [26].

Nowadays, much data has been accumulated on these diseases, allowing for the building of associative gene networks that describe the potential molecular mechanisms of interactions between the diseases. There are a number of resources in the world that allow reconstruction of such associative gene networks, for example, MetaCore [27], Ingenuity [28] and ANDSystem [29, 30]. In particular, using the developed by us ANDSystem tool, the following studies were performed: analysis of proteomic data on *Helicobacter pylori* infection [31]; analysis of the urine proteomic profile in control and under the influence of space flight factors [32]; analysis of tissue-specific gene knockout effect and the search for potential drug targets [33]; analysis of hepatitis C virus life cycle gene networks [34]; analysis of comorbid relations of bronchial asthma and tuberculosis [35], pre-eclampsia, diabetes and obesity [36], glaucoma [37]; search for novel candidate genes of susceptibility to tuberculosis [38].

The goal of this work was prioritization of candidate genes based on reconstruction and analysis of gene networks describing asthma and hypertension interactions. The associative network reconstructed in this work by ANDSystem [29, 30] details the interactions between genes/proteins that are linked to both asthma and hypertension, specifically including 205 genes and 9638 relations. It is worth noting that 69 genes from the network are related to apoptosis and 44 participate in central nervous system (CNS) functioning, suggestive of the important role of these processes for the formation of combined asthma and hypertension. From a ranked list of candidate genes, 10 can be highlighted as having the most priority. In particular, IL10, TLR4, and CAT had the highest priority across all examined scores, including standard methods of prioritization (Endeavor and ToppGene) as well as original methods that take into account the structure of the asthma/hypertension gene network and the associations of gene polymorphisms with the diseases. The predicted genes can be employed for planning of genotyping experiments.

## Methods

The reconstruction of associative gene networks of asthma and hypertension was carried out using the ANDSystem tool [29, 30]. The ANDSystem was developed to automatically analyse scientific publications in order to extract knowledge on the molecular genetic interactions and associations of proteins, genes, metabolites, drugs, and microRNAs with diseases, biological processes, drug side effects, and the phenotypes of various organisms. The ANDSystem knowledge base was built on the basis of a large-scale analysis of over 25 million abstracts of scientific papers presented in the PubMed database. In addition, information on molecular genetic interactions from different factual databases, such as IntAct, MINT, and others was integrated into ANDSystem. In total, more than seven million facts regarding molecular genetic interactions and associations are available in the ANDSystem knowledge base. In the current study we used ANDSystem version 2016. It is based on the analysis of all PubMed abstracts up to 2016, as well as information obtained from external databases that were available in 2016.

Enriched gene ontology (GO) biological processes were identified using the service DAVID 6.8 [39]. All settings were utilised in default mode.

To evaluate the centrality of vertices in the graphs of gene networks, the following functions from the network package of the Python programming language were used: “nx.degree_centrality” to calculate the degree centrality (DC), “nx.closeness_centrality” for calculating closeness centrality (CC), and “nx.betweenness_centrality” for betweenness centrality (BC) [40].

**Fig. 1:**
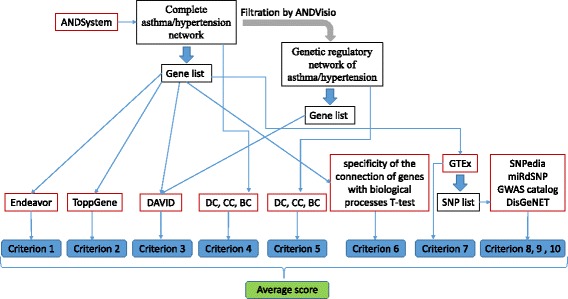
A general scheme for calculating criteria of gene prioritization

The scheme of the gene prioritization algorithm that includes 10 criteria is shown in Fig. 1. Criterion 1 was calculated using the Endeavor system for gene prioritization, version 3.71 (https://endeavour.esat.kuleuven.be/Endeavour.aspx) [41, 42]: Rank1_i_ = Rank(X_i_), where X – sorted list of genes according to Endeavor output, i – gene number. All settings used were in default mode. As the input for the test and training sets, the list of genes from the complete asthma/hypertension network was utilised.

Criterion 2 was calculated with the gene prioritization system, ToppGene (https://toppgene.cchmc.org/prioritization.jsp) [43, 44]: Rank2_i_ = Rank(X_i_), where X – sorted list of genes according to ToppGene output, i – gene number. All settings used were in default mode. The genes from the complete asthma/hypertension network were entered as the input, and the list of genes from the complete asthma/hypertension network, from which the analysed genes were excluded, was provided as a training set. Pearson’s correlation coefficient for criteria 1 and 2 ranks and its statistical significance were estimated using the Social Science Statistics resource (http://www.socscistatistics.com).

Criterion 3: involvement in the GO biological processes enriched in the complete and genetic regulatory networks of asthma/hypertension. This score was computed as Rank3_i_ = Rank(X_i_), where X – sorted list of genes according to N_i_ = N1_i_ + N2_i_, where N1_i_ – total number of enriched GO biological processes in complete networks of asthma/hypertension in which gene i was involved, N2 the same as N1 calculated for genetic regulatory networks of asthma/hypertension (see Additional file 1: Table S2).

Criterion 4: calculated for gene i as Rank4_i_ = Rank(X_i_), where X – sorted list of genes according to average measure of the value of DC, CC, and BC for each gene from complete network of asthma/hypertension.

Criterion 5 was calculated in the same way as Criterion 4 using genetic regulatory network of asthma/hypertension instead of complete network of asthma/hypertension.

Criterion 6: Rank6_i_ = Rank(X_i_), where X – sorted list of genes according to specificity of the connection of genes with biological processes associated with asthma and hypertension. To arrive at this score, at the first step, a list of biological processes connected with asthma and hypertension according to ANDSystem was constructed. The following types of interactions were considered: association, regulation, and treatment. For asthma, there were 357 linked biological processes and for hypertension, 338 processes. One hundred and eighteen biological processes were connected simultaneously with asthma and hypertension. Furthermore, all the biological processes presented in ANDSystem were divided into two groups. A test set that included 118 biological processes, associated simultaneously with asthma and hypertension, and a control set containing all the other 13,538 biological processes from ANDSystem. For each of the 205 genes/proteins, associated simultaneously with asthma and hypertension, interactions with biological processes were established using ANDSystem. The specificity of the connection between genes/proteins and the test set of biological processes simultaneously associated with asthma and hypertension was evaluated by applying the Student’s t-test. Student’s t-test was performed using the function stats.ttest_ind with the parameter equal_var = False, from the package, scipy.stats, in Python [45, 46]. A Bonferroni correction for multiple comparisons was conducted with the function, p.adjust (Y, “bonferroni”) of the “stats” package in the programming language R [47].

Criterion 7: Rank7_i_ = 1 if SNPs from list Y was present in gene i, otherwise Rank7_i_ was equal to maximal rank for list X (Rank7_i_ = 205), since the presence of such polymorphisms is of great importance for genotyping. List Y included all SNPs for each gene from X that were found in the eQTL gene region with the frequency of the minor allele in at least 5% in European population. A threshold of 5% allows to detect MAF polymorphisms with a high degree of probability using available genotyping arrays, thus it is often used in genomic analysis [48–50]. To calculate this score, the GTEx resource (http://www.gtexportal.org) [51] was consulted. It provides information on the variability of global expression of genes and SNPs affecting the level of gene expression. For the analysed genes, all SNPs localized in the region of the eQTL were taken from the database. Such SNPs may be relevant to the development of diseases [52–54]. Then, only the SNPs that altered the expression of the analysed genes in whole blood were selected. As the next step, for SNPs in the eQTL region, the prevalence of the minor allele among the European population was estimated. The analysis was carried out using the Ensembl database (http://www.ensembl.org) [55] based on the averaged frequencies of minor alleles for populations of European origin CEU (inhabitants of Western and Eastern Europe), GBR (Britain and Scotland), IBS (Spain), and TSI (Italy). In terms of further analysis, only SNPs that had a minor allele frequency of at least 5% in the European population were selected (for most of the found SNPs, the minor allele frequency was 20% or higher).

Criterion 8: Rank8_i_ = 1 if any gene i SNP associated with either asthma or hypertension was presented in list Y, otherwise Rank8_i_ was equal to 205.

Criterion 9: Rank9_i_ = 1 if in list Y for gene i an SNP associated with some disease comorbid to asthma or to hypertension was present, otherwise Rank9_i_ was equal to 205. Manual analysis of PubMed publications was conducted to generate a list of diseases with comorbidity to asthma and hypertension. To this end, for asthma, we manually examined 196 PubMed publications found by the query, “asthma comorbid diseases”, and filtered via the parameter, “Free full text”. For hypertension, 622 PubMed publications, obtained with the query, “hypertension comorbid diseases”, and filtered by the parameter, “Free full text”, were analysed.

Criterion 10: Rank10_i_ = 1 if in list Y for gene i SNP associated with any disease was present, except diseases specified for criterion 8 and criterion 9, otherwise Rank10_i_ was equal to 205. To calculate criteria 8-10, information on the associations of SNPs with diseases was extracted from the databases, SNPedia [56], miRdSNP [57], GWAS catalog [58], and DisGeNET [59, 60]. It was considered that a polymorphism was associated with a disease if this information was found in at least in one of the databases.

For each gene, the final score was computed as the average value of ranks formulated according to criteria 1-10.

An independent evaluation of genes selected according to these criteria was carried out by analyzing the normalized frequency of their mentioning in PubMed together with the “comorbid” or “comorbidity” terms. The frequency of references (F) was calculated as the number of PubMed abstracts in which the gene name was mentioned together with “comorbid” or “comorbidity” divided by the total number of PubMed abstracts where the gene was mentioned. An analysis of the enrichment of the list of top genes by genes, which are often mentioned in the discussion of comorbid states of various diseases, was carried out by comparing the average frequencies F calculated for a set of top genes with a complete list of genes, according to the Mann-Whitney test, estimated by the function «mannwhitneyu» from the package «scipy.stats» of Python [45, 46].

The formation of lists of genes associated with apoptosis for the GO category “apoptotic process” (GO: 0006915) along with genes involved in the functioning of the CNS for GO categories “neurotransmitter secretion” (GO:0007269), “neurogenesis” (GO:0022008), “multicellular organismal response to stress” (GO:0033555), “social behaviour” (GO:0035176), “cognition” (GO:0050890), “response to antipsychotic drug” (GO:0097332), and “response to psychosocial stress” (GO:1,990,911) was performed using the AmiGO 2 database [61, 62] available at http://amigo.geneontology.org/. Only the human genes involved in the analysed GO categories were selected.

The statistical significance of the differences between the centrality of the apoptosis genes and the rest of the genes of the analysed networks was estimated by the function “stats.ttest_ind” with the parameter equal_var = False from the package “scipy.stats” of Python [45, 46]. Similarly, the statistical significance of the differences between the centrality indices of the CNS genes and the remaining genes of the analysed networks was evaluated.

## Results and discussion

### Associative gene networks of asthma and hypertension

In order to find the molecular genetic mechanisms underlying the development of asthma and hypertension, we compiled a list of 755 genes/proteins associated with asthma and 713 genes/proteins associated with hypertension according to ANDSystem (Additional file 2: Table S1). The gene network of asthma included 62,603 interactions between 755 genes and 751 proteins, including 2402 genetic regulations, 920 activity regulations, 79 degradation regulations, 625 transport regulations, 2594 protein-protein interactions, 751 expression links, 75 co-expression links, 159 chemical transformations, and 54,998 associative interactions. In ANDSystem associative interaction is a special type of interactions reflecting any types of relations between two objects including listed above.

The gene network of hypertension included 45,479 interactions between 713 genes and 710 proteins, including 1373 genetic regulations, 709 activity regulations, 71 degradation regulations, 423 transport regulations, 1905 protein-protein interactions, 708 expression links, 31 co-expression links, 165 chemical transformations, and 40,094 associative interactions. There are suggestions in the literature that putative candidate genes for the development of comorbid conditions between a pair of diseases are genes simultaneously associated with both diseases [63–65]. Previously, for such diseases as bronchial asthma and tuberculosis, we showed the potential role of genes concurrently linked with both of them in the pathogenesis of their comorbid relationships [35]. The network of interactions between genes and proteins, associated simultaneously with asthma and hypertension (complete asthma/hypertension network), constructed by intersection of the asthma and hypertension networks, included 85 genes, 201 proteins, and 9638 interactions of 17 types. It should be noted that the complete asthma/hypertension network included the same types of interactions as the separate networks of asthma and hypertension: 345 genetic regulations, 347 activity regulations, 25 degradation regulations, 262 transport regulations, 554 protein-protein interactions, 84 expression links, three co-expression links, 45 chemical transformations, and 7973 associative interactions. In summary, none of the types of interactions disappeared upon building up complete asthma/hypertension network.

The enriched GO biological processes (*p*-value < 0.01 with FDR correction) for genes/proteins associated with asthma were identified with the DAVID 6.8 system. It was observed that among the most significant GO biological processes were inflammatory response, immune response, response to hypoxia, regulation of T cell proliferation, neutrophil chemotaxis, platelet degranulation, and regulation of interleukin production (Additional file 1: Table S2). For genes/proteins associated with hypertension, among the most significant GO biological processes were regulation of blood pressure, response to drug, response to hypoxia, inflammatory response, aging, regulation of vasodilation, response to insulin, and angiogenesis (Additional file 1: Table S2). Among the most highly enriched GO biological processes for genes/proteins associated simultaneously with asthma and hypertension (complete asthma/hypertension network) were response to hypoxia, positive regulation of nitric oxide biosynthetic process, regulation of blood pressure, aging, inflammatory response, and negative regulation of apoptotic process (Additional file 1: Table S2). These processes may be the most significant for the comorbid relationship between asthma and hypertension.

Among the GO biological processes that were enriched for the asthma network and not featured in the list of enriched processes for the complete asthma/hypertension network, were microglial cell activation, regulation of interleukin production, positive regulation of tissue remodelling, and regulation of cytokine secretion. Those GO biological processes enriched only for the hypertension network were angiotensin maturation, regulation of the force of heart contraction, response to insulin, vasoconstriction, cholesterol homeostasis, and negative regulation of feeding behaviour. Such processes, apparently, are more pertinent to the mechanisms of development of individual asthma or hypertension. The GO biological processes, removal of superoxide radicals, protein kinase B signalling, positive regulation of isotype switching to IgG isotypes, and positive regulation of peptidyl-serine phosphorylation, were enriched only for the complete asthma/hypertension network and not for the individual asthma or hypertension networks.

**Fig. 2:**
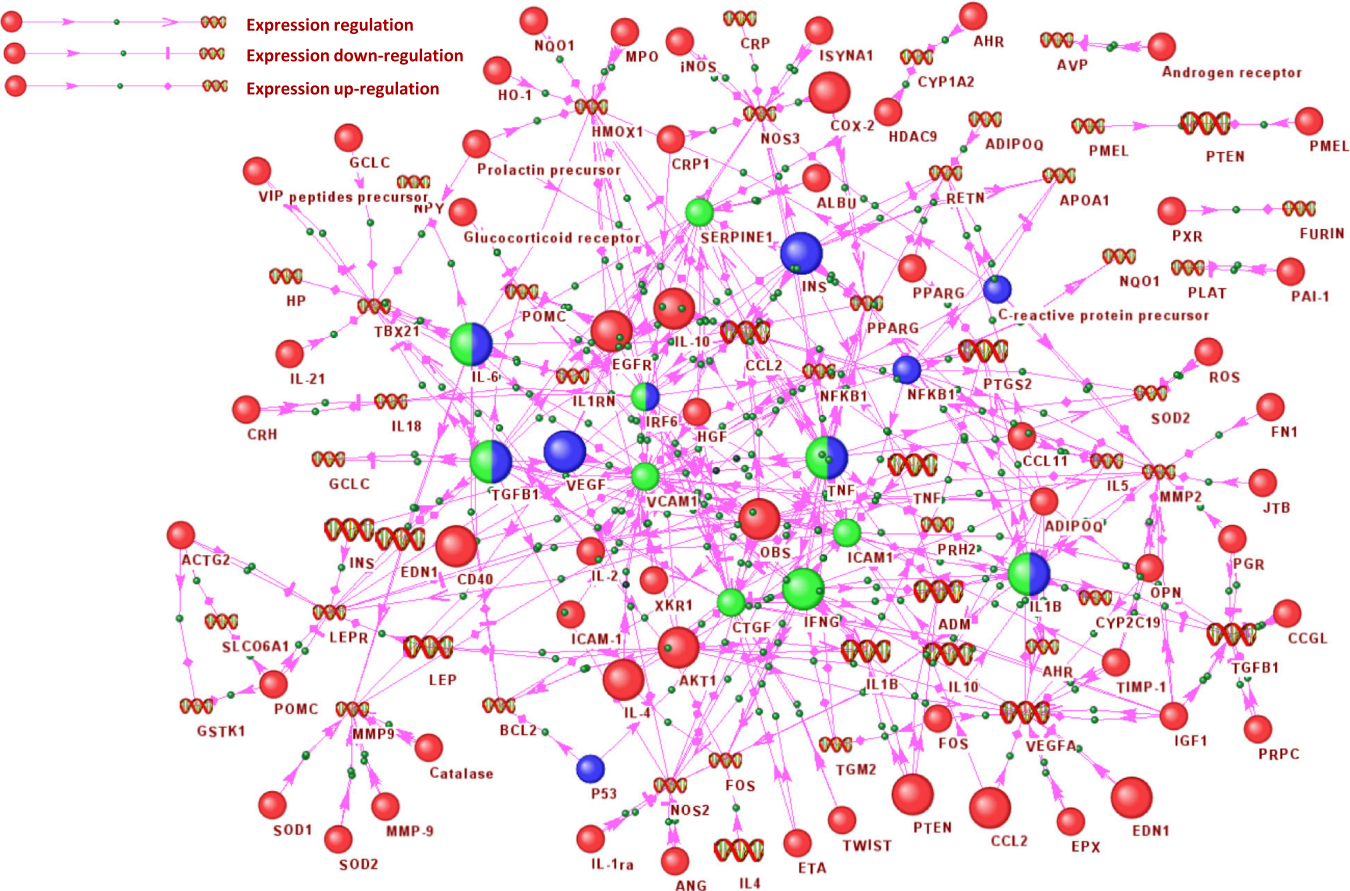
Genetic regulatory network of asthma/hypertension. Proteins are presented by circles and genes are represented by DNA helix. Proteins involved in a large number of enriched GO biological processes (more than 20) for both the complete and genetic regulatory network of asthma/hypertension are shown with large icons. The top 10 proteins with the highest value of betweenness centrality in the complete asthma/hypertension network are highlighted in *blue*; in the genetic regulatory network of asthma/hypertension, in *green*; in both networks, in two-color *green*/*blue*. Picture was done using the ANDVisio program, which is a part of ANDSystem, and gene/protein notations are given according to ANDVisio output

It is known that genetic regulation is paramount for the genetic variability in diseases across patients [66–68]. The genetic regulatory network of asthma/hypertonia, including interactions between genes involved in expression regulation, expression up-regulation, and expression down-regulation, is portrayed in Fig. 2. This network contains 52 genes, 68 proteins, and 345 interactions. At the same time, from the Fig. 2 it can be seen, that general regulatory network can be divided into at least five subnetworks, including four small subnetworks containing from 2 to 3 participants (for example, PXR protein → Furin gene). These subnetworks appeared to be unconnected with the core of the regulatory network, because in ANDSystem they were connected only by associative interaction type. It was interesting to evaluate the enrichment of GO biological processes for genes/proteins from the genetic regulatory network of asthma/hypertension (Additional file 1: Table S2). It turned out that for this network seven new enriched GO biological processes were identified (response to heat, positive regulation of ERK1 and ERK2 cascade, embryo implantation, positive regulation of B cell proliferation, glucose homeostasis, positive regulation of JAK-STAT cascade, and defence response to protozoan) and these were not significant within the whole asthma/hypertension network. Among the GO processes that were simultaneously significant for the complete and genetic regulatory network of asthma/hypertension were negative regulation of apoptotic process, positive regulation of nitric oxide biosynthetic process, inflammatory response, and several others.

One of the most central regulatory nodes for both the complete and genetic regulatory network of asthma/hypertension is TNF-α (Fig. 2). We observed that this gene is involved in a large number of enriched GO biological processes. For example, it participates in inflammatory response, immune response, the positive regulation of the nitric oxide biosynthetic process, positive regulation of NF-kappaB transcription factor activity, and is closely related to apoptosis. It is known that the level of TNF-α is elevated in both asthma and hypertension patients. The levels of TNF-α are increased in the airway tissues of asthmatic subjects and TNF-α expression has been seen to be up-regulated in alveolar macrophages, mast cells, and bronchial epithelial cells [69, 70]. TNF-α was also found to be higher in concentration in chronic inflammatory states, such as hypertension, and is implicated in both increases and decreases in blood pressure [71]. It is interesting that the TNF-α, which has a high centrality, turned out to be connected in the regulatory network with NF-kappaB, which also has a high centrality value (Fig. 2). It is known that biological networks are characterized by a low degree of assortativity, i.e. vertexes with a large number of connections are rarely connected with each other [72]. Thus, the uncovered interactions between TNF-α and NF-kappaB may indicate the special role of this connection for the comorbid state of asthma and hypertension. Further, TNF-α can activate the expression of NF-kappaB and increase its activity [73]. It was previously demonstrated that in both asthma and hypertension, the activity of NF-kappaB is enhanced [74–77]. Figure 2 illustrates that, in turn, NF-kappaB is able to reduce the level of expression of the apolipoprotein A1 (apoA-1) gene [78]. There are data suggesting that in cases of hypertension, the level of apoA-1 is diminished [79, 80]. With this, in asthma patients, the level of apoA-1 in bronchoalveolar lavage fluid was significantly lower than in healthy controls [81, 82]. ApoA-1 has a specific role in lipid metabolism, and is the major component of HDL particles in blood [83]. It is interesting to note that the apoA-1 gene is involved in the GO category neurogenesis (GO:0022008), related to the CNS. Thus, it can be seen that, the various biological processes featured in the pathogenesis of asthma and hypertension, as well as their comorbid development, including apoptosis and CNS processes, can be mediated through regulatory interactions.

### Prioritization of candidate genes

Gene prioritization is a task of many studies aimed at candidate gene identification. Among the existing tools for gene prioritization, there are Endeavour [41, 42], ToppGene [43, 44], and DIR [84]. These programs allow one to rank a test set of genes based on a training set of genes according to certain criteria characterizing the proximity of genes from the test set to the genes from the training set. The methods of these resources employ properties of the vertices of gene network graphs, genetic information (co-localization in the genome), functional properties of genes (involvement in the same GO categories), etc. To search for candidate genes that might have an important part in the molecular genetic mechanisms of asthma and hypertension comorbidity, here, we utilised the Endeavor (criterion 1) and ToppGene (criterion 2) programs. Additionally, to take into account the structure of the gene network, describing the interactions between asthma and hypertension, as well as polymorphisms in the genes associated with the studied diseases, criteria 3-10 were used. In particular, information about polymorphisms was used in criteria 7-10 in the following way: all genes with known polymorphisms had a minimal rank (equal to 1), while the rank of remaining genes had maximal value (equal to 205). It allowed to provide criteria 7-10 with a more weight compared to other criteria. We believe that the presence of polymorphisms in the studied genes is important for the development of comorbidity. The values of the listed scores for the top ten genes from the complete asthma/hypertension network are shown in Table 1.

**Table 1:** Top 10 genes with the highest priority according to average rank

Gene name	Rank 1	Rank 2	Rank 3	Rank 4	Rank 5	Rank 6	Rank 7	Rank 8	Rank 9	Rank 10	Average rank
IL10	44	140	6	14	26	9	1	1	1	1	24.3
TLR4	11	92	17	43	92	29	1	1	1	1	28.8
CAT	59	78	11	31	71	37	1	1	1	1	29.1
NFKB1	3	26	13	5	16	8	1	205	205	1	48.3
AKT1	9	64	4	20	20	18	1	205	205	1	54.7
ADRB2	78	82	28	166	92	155	1	1	1	1	60.5
ICAM1	26	86	11	30	3	44	1	205	205	1	61.2
CST3	112	105	25	117	92	117	1	205	1	1	77.6
POMC	93	85	23	59	53	51	1	205	205	1	77.6
SPP1	25	45	26	44	30	5	1	205	205	205	79,1

According to criterion 1, among the top ten most important genes/proteins, sorted by the “*P*-value” indicator, were TNF, FN1, NFKB1, TGFB1, APOA1, EGFR, MMP9, RELA, AKT1, and PLAT (Additional file 3: Table S3). For criterion 2 the list of the top ten genes/proteins, ranked according to the “Average Score” indicator, included FURIN, PTGS2, TIMP1, VCAM1, NPY, CALM3, HP, RAN, AOC1, and IL4 (Additional file 4: Table S4). The correlation coefficient of the ranks, calculated according to criteria 1 and 2, was *R* = 0.548 with a *p*-value < 10^− 5^.

Criterion 3 suggested that for both the complete and genetic regulatory network of asthma/hypertension, IL6 was involved in the greatest number of over-represented GO biological processes - 24 and 27 processes, respectively (Additional file 5: Table S5). Ranking by criterion 3 demonstrated that for 18 genes/proteins (IL6, TGFB1, TNF, IL1B, AKT1, CCL2, IL4, IL10, EGFR, LEP, PTGS2, PTEN, EDN1, VEGFA, IFNG, ADM, CD40, INS), the total number of GO biological processes in which these genes/proteins participated with respect to the complete and genetic regulatory network of asthma/hypertension was more than 20 (Fig. 2).

According to criteria 4 and 5, it turned out that the genes/proteins with the highest centrality index for both the complete and genetic regulatory network of asthma/hypertension were IL6, TGFB1, TNF, IL1B, and IRF6. The highest centrality index for just the complete network was for genes INS, NFKB1, VEGFA, TP53, and CRP, and for the genetic regulatory network of asthma/hypertension, genes VCAM1, ICAM1, CTGF, IFNG, SERPINE1 (Additional file 6: Table S6).

According to criterion 6, 154 genes/proteins are specifically associated with the test set of biological processes with a Bonferroni corrected *p*-value < 0.01 (Additional file 7: Table S7). Among the genes most significantly associated with the test set were TNF, INS, IL6, LEP, SPP1, VEGFA, IGF1, NFKB1, IL10, and TGFB1.

**Fig. 3:**
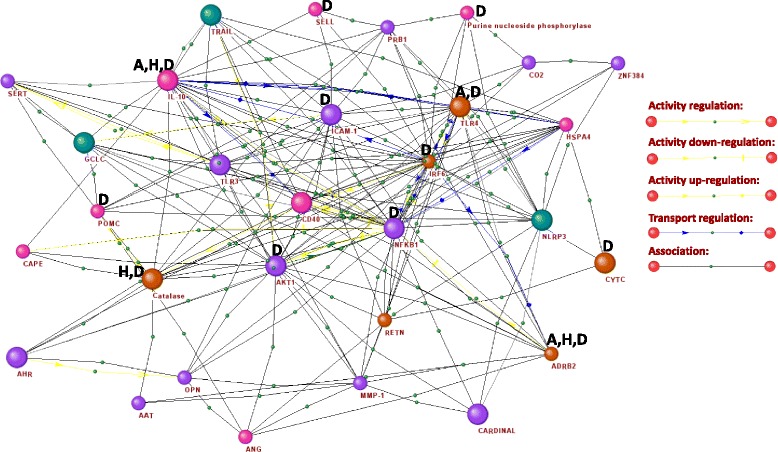
Network of interactions between genes/proteins that had SNPs determined by criteria 7-10. Cyan colour - a small number of polymorphisms (0.01-0.099 SNPs per 1000 nucleotides), purple colour - a moderate number of polymorphisms (0.1-0.9 SNPs per 1000 nucleotides), pink colour - a high number of polymorphisms (1.0-4.9 SNPs per 1000 nucleotides), orange colour – a very high number of polymorphisms (5.0-31.1 SNPs per 1000 nucleotides). D — gene polymorphisms associated with various diseases; A — gene polymorphisms associated with asthma; H — gene polymorphisms associated with hypertension. Large icons indicate genes/proteins associated with apoptosis. Picture was done using the ANDVisio program

Criterion 7 showed that of the 205 analysed genes, 30 genes had SNPs found in the eQTL. Moreover, we revealed that there were 1425 SNPs (Additional file 8: Table S8). The highest number of SNPs (more than seven per 1000 nucleotides) was observed for genes ADRB2, TLR4, CST3, IRF6, CAT, and RETN (Fig. 3). Of these, ten polymorphisms in the ADRB2, IL10 and TLR4 genes were associated with asthma, and seven polymorphisms in ADRB2, IL10 and CAT were linked to hypertension (Fig. 3). These genes had the highest priority according to criterion 8. The eight polymorphisms in genes ADRB2, IL10, CAT, TLR4, and CST3 were linked with any disease comorbid to asthma or hypertension (e.g., diabetes mellitus, arthritis, myocardial infarction, kidney diseases, diabetic nephropathy). According to criterion 9, genes ADRB2, IL10, CAT, TLR4, and CST3 had the highest priority. Analysis of the associations of the pertinent SNPs with other diseases uncovered 51 SNPs in 12 genes (Additional file 9: Table S9). Thus, according to criterion 10, the highest priority was given to genes ADRB2, IL10, CAT, TLR4, ICAM1, IRF6, AKT1, CST3, NFKB1, PNP, POMC, and SELL.

In reviewing the average rank (Additional file 10: Table S10), ten genes (IL10, TLR4, CAT, NFKB1, AKT1, ADRB2, ICAM1, POMC, CST3 and SPP1) had the highest priority (Table 1). It appeared that all genes, presented in Table 1, except CST3, were associated with asthma and/or hypertension, according to the OMIM [85] and MalaCards [86] databases. However, the associations of this gene with asthma and hypertension are discussed in the literature [87–89].

An independent analysis of co-occurrence of genes with “comorbid” or “comorbidity” terms showed that these top ten genes (F = 0.023) are more frequently (*p*-value < 0.05) mentioned together with these terms, compared to the total set of 205 genes (F = 0.006) from the complete regulatory network of asthma/hypertension. Thus, this may indicate a potentially important role for the comorbid state of asthma and hypertension. In particle, among these top genes TLR4 and ADRB2 (2nd and 6th place in Table 1) are directly discussed in the literature in the context of the comorbidity of asthma and hypertension [21–24, 26]. TLR4 is involved in activation of the innate immune system via the NF-κB signalling pathway along with the up-regulation of inflammatory cytokine production. With this, the expression of TLR4 was observed to be up-regulated in asthma [90]. Up-regulation of TLR4 has also been observed after myocardial infarction and inhibition of TLR4 decreases blood pressure [91]. The ADRB2 gene encodes a beta-2 adrenergic receptor mediating catecholamine-induced activation of adenylate cyclase via G proteins. ADRB2 is a known drug target to treat asthma [92, 93] and a number of SNPs in this gene are associated with asthma [94, 95] and hypertension [96, 97].

Other interesting genes are IL10 and CAT, which had 1st and 3rd places in Table 1, respectively. IL-10 is an anti-inflammatory cytokine derived from CD4+ T-helper type 2 (T(H2)) cells, and in cases of asthma, a relative underproduction of IL-10 from alveolar macrophages was reported [98]. During asthma, IL-10 can inhibit eosinophilia via suppression of IL-5 and GM-CSF, regulate eosinophil apoptosis, and down-regulate IL-1. In addition, IL-10 can suppress nitric oxide production, an important component of airway inflammation [99]. Up-regulation of IL-10 was also demonstrated to normalize blood pressure and endothelial function [100, 101].

Superoxide anion and hydrogen peroxide were found in higher concentrations in both asthma and hypertension patients compared with controls [102, 103]. Catalase (CAT) is an enzyme that catalyses the decomposition of hydrogen peroxide to water and oxygen, and it was observed that catalase overexpression can prevent hypertension [104] and that catalase activity was enhanced during treatment of asthma [105, 106].

### Apoptosis in asthma/hypertension gene network

Apoptosis is one of the processes that features most prominently in various diseases. It is actively studied in the pathogenesis of asthma and hypertension [107, 108], and it has been suggested that deregulation of apoptosis in activated T cells and eosinophils are involved in the development of airway inflammation in asthma [109, 110]. With regard to hypertension, there is evidence of increased apoptosis in whole organs [111, 112]. Despite the fact, that apoptosis is a well-studied regulatory network, the role of apoptosis genes in the structure of gene networks of these two diseases requires further clarification. A total of 1873 genes are implicated in the apoptotic process (GO: 0006915) according to the AmiGO database [61, 62]. In the complete and genetic regulated gene network of asthma/hypertension, 69 and 48 genes of apoptosis were included, which are 34 and 53% of all the genes of the analysed networks, respectively. Analysis of the centrality of these genes in the complete network showed that the average DC value is 0.299, CC value was 0.579, and BC value was 0.0064. Additionally, it appeared that these indicators were statistically significant (*p*-value < 10^− 4^) more than those for the other genes of the complete network (DC - 0.147, CC - 0.515, BC - 0.0018). In the genetic regulatory network, the centrality of the apoptosis genes (DC - 0.038, CC - 0.296, BC - 0.023) also exceeded the CC (BC - 0.025, CC - 0.256, DC - 0.014), although no statistically significant differences were noted.

**Fig. 4:**
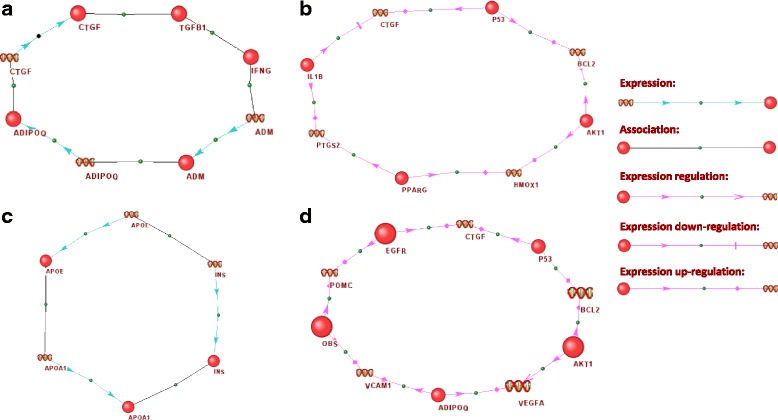
Fundamental cycles revealed in complete and genetic regulatory asthma/hypertension networks associated with apoptosis and CNS. **a** Cycle from the complete asthma/hypertension network, which includes just the genes/proteins associated with apoptosis (CTGF, ADM, ADIPOQ, TGFB1, and IFNG). **b** Cycle from the genetic regulatory network of asthma/hypertension, which includes just the genes/proteins associated with apoptosis (CTGF, BCL2, HMOX1, PTGS2, P53, AKT1, PPARG, and IL1B). **c** Cycle from the complete asthma/hypertension network, which includes just the genes/proteins associated with the CNS (APOE, INS, and APOA1). **d** Cycle from the genetic regulatory asthma/hypertension network, which includes five participants associated with the CNS (genes: VEGFA, BCL2; proteins: AKT1, OBS, EGFR). Proteins are presented by circles and genes are represented by DNA helix. Picture was done using the ANDVisio program

To further assess the structural role of the apoptosis genes in the graphs of the complete and genetic regulatory networks of asthma/hypertension, we evaluated the fundamental cycles using the “Find fundamental rings” function of ANDSystem [29, 30]. Fundamental cycles are those that form the basis of a cyclic space of a graph, that is, any cycle of a graph can be represented by the sum of fundamental cycles. In the complete and genetic regulatory networks of asthma/hypertension, 9354 and 230 fundamental cycles were found, respectively. It turned out that among them, 9201 and 219 cycles contained at least one gene/protein associated with apoptosis, respectively. Further, the number of cycles that featured only the genes of apoptosis was 191 for the complete network and 31 for the genetic regulatory network of asthma/hypertension. In particular, the cycle of maximum length for the complete network (Fig. 4a) among all cycles, including only the apoptotic genes, consisted of three genes (CTGF, ADM, ADIPOQ) and five proteins (CTGF, ADM, ADIPOQ, TGFB1, IFNG). In this cycle, the protein, IFNG, differentially regulates TGF-beta1 [113] and the up-regulated secretion of TGF-beta is accompanied by down-regulation of IFN-gamma [114]. IFN-gamma and IL-1beta can induce expression of the ADM gene in ARPE-19 cells [115]. As well, it is known that plasma ADM protein levels are related to SNP rs182052 in the ADIPOQ gene [116]. In turn, the ADIPOQ protein can down-regulate CTGF mRNA and proteins [117] and TGFB1 can elevate CTGF transcript levels [118]. For the genetic regulatory network, a similar cycle (Fig. 4 B) included four genes (CTGF, BCL2, HMOX1, PTGS2) and four proteins (P53, AKT1, PPARG, IL1B). In this cycle, it could be seen that the p53 protein can bind the Bcl2 protein to form a complex that influences apoptosis regulation [119], and moreover p53 was shown to induce temperature-dependent decrease in the expression of the bcl-2 gene [120]. Bcl-2 gene expression is also regulated by activation of Akt [121]. Subsequently, Akt can regulate the expression of the HMOX1 gene [122]. The expression of HMOX1 gene can be up-regulated by activation of PPARG [123]. The activation of PPARG can also suppress expression of the PTGS2 gene [124]. Further, the expression of the PTGS2 gene can be up-regulated by IL1B [125, 126]. In turn, IL1B can significantly suppress CTGF gene expression [127], of which expression can be induced by p53 protein [128].

Along with the important role of apoptosis genes in the structure of the asthma/hypertension gene network, the apoptosis genes had high priority in Additional file 10: Table S10. Their average rank was 108.4, which is less statistically significant (*p*-value < 10^− 10^) than the average rank throughout the remainder of the Table (127.3). Thus, among the ten top genes, seven were associated with apoptosis (Table 1). Such high enrichment in Table 1 can be related to their high centrality and participation in a large number of fundamental cycles of the asthma/hypertension gene network. Among the genes of apoptosis and anti-apoptosis network with the highest priority according to the total score, one can distinguish IL10, TLR4, CAT, NFKB1, AKT1, ICAM1, and CST3. As mentioned before, IL-10 is an anti-inflammatory cytokine and can induce macrophage apoptosis [129, 130]. TLR4 stimulation also induces apoptosis [131] and can mediate neuronal apoptosis [132]. Moreover, TLR4 is necessary for the immunological mechanism of apoptosis [133]. Meanwhile, CAT was shown to inhibit apoptosis in different cells, including T cells [134, 135]. NF-kappaB controls cytokine production, cell survival, and can block apoptosis [136]. Suppression of NF-kappaB also induces apoptosis [137]. Akt can suppress apoptosis via activation of the RelA/p65 subunit of NF-κB [138] and phosphorylation-dependent cleavage of Akt can influence apoptosis in neural cells [139]. ICAM-1 is able to influence release of various inflammatory cytokines and reactive oxygen species and because of that, its involvement is notable in apoptosis regulation [140]. Induced expression of ICAM-1 leads to participation in inhibition of apoptosis [141]. Cystatin C (CST3) expression in vascular wall smooth muscle cells is diminished in certain vascular diseases, and it is known that CST3 can bring about apoptosis [142].

### Genes involved in functioning of the central nervous system in the asthma/hypertension gene network

At present, there is increased interest among researchers in the problem of the effect of various pathological processes on the CNS, including inflammation, asthma, and hypertension [143–145]. It is discussed in the literature that hypertension and/or hypoxia can activate neurogenesis as a response to neuronal loss induced by these factors [146, 147]. There is an evidence that certain elements of sympathetic neurotransmission can be activated during hypertension [148]. In keeping with this, it has been demonstrated that hypertension can lead to the memory loss as well as impair learning [147, 149] and cognition [150]**.** Interestingly, in Guo et al. [151], it was shown that chronic asthma can affect cognitive functions and impact synaptic transduction [152] and neurogenesis [153, 154]. It has also been discussed that psycho-social stress and psychological factors can play an important role in bronchial asthma [155, 156].

In order to clarify the role of genes involved in the functioning of the CNS in the asthma/hypertension gene network, GO biological processes associated with asthma and hypertension and involved in the functioning of the CNS were selected using ANDSystem. It was found that of 357 biological processes associated with asthma, there were six terms of the CNS (neurogenesis, cognition, neurotransmitter secretion, response to psychosocial stress, social behaviour, and response to antipsychotic drug), and among the 338 of biological processes associated with hypertension, there were four such terms (neurogenesis, cognition, neurotransmitter secretion, and multicellular organismal response to stress). A small number of GO terms associated with asthma and hypertension associated with the CNS can be explained by the lack of knowledge surrounding this matter. This means a study on the relationship of CNS genes with these diseases is warranted. The following GO terms were considered: neurotransmitter secretion (GO:0007269), neurogenesis (GO:0022008), multicellular organismal response to stress (GO:0033555), social behaviour (GO:0035176), cognition (GO:0050890), response to antipsychotic drug (GO:0097332), and response to psychosocial stress (GO:1,990,911).

A total of 2017 genes were elicited with these seven GO terms according to the AmiGO database [61, 62]. In the complete and genetic regulatory gene networks of asthma/hypertension, 44 CNS genes out of 205 genes and 27 CNS genes out of 91 genes, respectively, were found.

Analysis of the complete network showed that the centrality of these genes (DC - 0.277, CC - 0.573, BC - 0.005), as well as of the apoptosis genes, statistically significantly exceeded the average centrality of the other genes from the network (DC - 0.177, CC - 0.527, BC - 0.003), with a *p*-value < 0.05. Similarly, as for apoptosis, the average centrality of the CNS genes (DC - 0.0336, CC - 0.3, BC - 0.0194) surpassed the average gene centrality in the genetic regulatory network (BC - 0.0315, CC - 0.267, DC - 0.0193). It is noteworthy that among the 44 genes of the CNS, there were 26 genes of apoptosis. These genes possessed increased centrality within the CNS gene grouping. Thus, apoptosis can have a significant effect on the functioning of the CNS sub-network in the asthma/hypertension gene network.

The analysis of the fundamental cycles showed that in the complete and genetic regulatory network of asthma/hypertension, there were 8999 and 199 fundamental cycles, respectively, that contained at least one gene/protein associated with the CNS. It should be acknowledged that for apoptosis, for both network fundamental cycles, only the included genes of this process were found. In the case of the CNS, the fundamental cycles consisting of CNS genes were detected only in the complete network, numbering at 59. In particular, the cycle of maximum length for a complete network, including only CNS genes, consisted of genes APOE, INS, and APOA1, and proteins APOE, INS, and APOA1 (Fig. 4 C). In this cycle, it was demonstrated that INS initiated the synthesis of apoE [157, 158] and APOA1 gene expression [159, 160]. In turn, apoA1 protein may enhance local secretion and accumulation of apoE and hence influence anti-atherogenic processes [161].

With respect to the fundamental cycles found in the genetic regulatory network of asthma/hypertension, the maximum proportion of CNS genes did not go beyond 50%. For example, in this fundamental cycle, there were five genes (VEGFA, BCL2, VCAM1, POMC, CTGF), five proteins (AKT1, OBS, EGFR, P53, ADIPOQ), two CNS genes (VEGFA, BCL2), and three CNS proteins (AKT1, OBS, EGFR) (Fig. 4 D). In this cycle, as in the cycle portrayed in Fig. 4 B, p53 protein interacted with Bcl-2 [119] and can induce the expression of the CTGF gene [128]. Bcl-2 gene expression is regulated by activation of Akt [121] and Akt influences regulation of VEGF-A expression [162]. VEGF-A expression is promoted by ADIPOQ through the ADIPOQ receptor, AdipoR [163]. ADIPOQ and OBS are able to induce VCAM-1 expression [164]. Increased OBS concentrations are linked with reduced POMC mRNA expression [165]. Of interest is that EGFR has been demonstrated to regulate expression of the POMC gene [166] and stimulate expression of CTGF [167].

## Conclusion

Computer reconstruction and analysis of gene networks makes it possible to put forward hypotheses about the molecular mechanisms of diseases. It also seems to be an effective tool for studying the complex interrelationships between diseases as comorbid conditions. The reconstructed asthma/hypertension gene network, which describes the potential molecular-genetic interactions between the two diseases, included 205 genes/proteins. Analysis of the sub-networks of apoptosis and the CNS showed that the genes of the CNS, like the genes implicated in apoptosis, are represented to a large extent in the asthma/hypertension network (69 and 44 genes, respectively) and can play an important role in its structure. Therefore, they can be important for the development of the comorbid condition of these two diseases.

Based on standard methods of prioritization, as well as original criteria that utilise the structure of the asthma/hypertension gene network, 10 candidate genes for genotyping and searching for drug targets have been proposed. The highest priority was given to the genes IL10, TLR4, and CAT, which occupy an important position in the immune system and apoptosis. It appeared that apoptotic genes had a special place in this top list of candidate genes, which was highly enriched with the genes of apoptosis. CNS genes were also present in the top list. We believe that the role of CNS genes in the pathology of these diseases and their comorbid conditions are not yet fully understood and merit close attention in the future via additional experimental studies.

## Additional files

Additional file 1: Table S2. Results of gene ontology enrichment analysis. (XLSX 280 kb)

Additional file 2: Table S1. Lists of genes associated with asthma and hypertension. (XLSX 105 kb)

Additional file 3: Table S3. Criterion 1 results. (XLSX 59 kb)

Additional file 4: Table S4. Criterion 2 results. (XLSX 41 kb)

Additional file 5: Table S5. Criterion 3 results. (XLSX 18 kb)

Additional file 6: Table S6. Criteria 4 and 5 results. (XLSX 36 kb)

Additional file 7: Table S7. Criterion 6 results. (XLSX 23 kb)

Additional file 8: Table S8. Criterion 7 results. (XLSX 63 kb)

Additional file 9: Table S9. Criteria 8-10 results. (XLSX 20 kb)

Additional file 10: Table S10. Total score results. (XLSX 34 kb)

## Abbreviations

BC: Betweenness centrality
CC: Closeness centrality
CNS: Central nervous system
DC: Degree centrality
eQTL: Expression quantitative trait loci
FDR: False discovery rate
GO: Gene Ontology
SNP: Single-nucleotide polymorphism
